# IgM in human immunity to *Plasmodium falciparum* malaria

**DOI:** 10.1126/sciadv.aax4489

**Published:** 2019-09-25

**Authors:** M. J. Boyle, J. A. Chan, I. Handayuni, L. Reiling, G. Feng, A. Hilton, L. Kurtovic, D. Oyong, K. A. Piera, B. E. Barber, T. William, D. P. Eisen, G. Minigo, C. Langer, D. R. Drew, F. de Labastida Rivera, F. H. Amante, T. N. Williams, S. Kinyanjui, K. Marsh, D. L. Doolan, C. Engwerda, F. J. I. Fowkes, M. J. Grigg, I. Mueller, J. S. McCarthy, N. M. Anstey, J. G. Beeson

**Affiliations:** 1 Burnet Institute for Medical Research and Public Health, Melbourne, Victoria, Australia.; 2 Global and Tropical Health Division, Menzies School of Health Research, Darwin, Northern Territory, Australia.; 3 QIMR Berghofer Medical Research Institute, Brisbane, Queensland, Australia.; 4 Department of Medicine, University of Melbourne, Melbourne, Victoria, Australia.; 5 Department of Immunology and Pathology, Monash University, Melbourne, Victoria, Australia.; 6 Charles Darwin University, Darwin, Northern Territory, Australia.; 7 Infectious Diseases Society Sabah-Menzies School of Health Research Clinical Research Unit, Queen Elizabeth Hospital, Kota Kinabalu, Sabah, Malaysia.; 8 Gleneagles Hospital Kota Kinabalu Sabah, Malaysia.; 9 Australian Institute of Tropical Health and Medicine, James Cook University, Cairns, Queensland, Australia.; 10 Kenya Medical Research Institute (KEMRI), Centre for Geographic Medicine, Coast, KEMRI-Wellcome Trust Research Programme, Kilifi, Kenya.; 11 Imperial College, London, UK.; 12 Nuffield Department of Medicine, University of Oxford, Oxford, UK.; 13 Centre for Epidemiology and Biostatistics, Melbourne School of Population and Global Health, The University of Melbourne, Melbourne, Victoria, Australia.; 14 Department of Epidemiology and Preventive Medicine, Department of Infectious Diseases, Monash University, Melbourne, Victoria, Australia.; 15 Walter and Eliza Hall Institute, Melbourne, Victoria, Australia.; 16 Department of Medical Biology, University of Melbourne, Melbourne, Victoria, Australia.; 17 Department of Parasites and Insect Vectors, Institute Pasteur, Paris, France.; 18 The University of Queensland, Brisbane, Queensland, Australia.; 19 Department of Microbiology and Central Clinical School, Monash University, Melbourne, Victoria, Australia.

## Abstract

Most studies on human immunity to malaria have focused on the roles of immunoglobulin G (IgG), whereas the roles of IgM remain undefined. Analyzing multiple human cohorts to assess the dynamics of malaria-specific IgM during experimentally induced and naturally acquired malaria, we identified IgM activity against blood-stage parasites. We found that merozoite-specific IgM appears rapidly in *Plasmodium falciparum* infection and is prominent during malaria in children and adults with lifetime exposure, together with IgG. Unexpectedly, IgM persisted for extended periods of time; we found no difference in decay of merozoite-specific IgM over time compared to that of IgG. IgM blocked merozoite invasion of red blood cells in a complement-dependent manner. IgM was also associated with significantly reduced risk of clinical malaria in a longitudinal cohort of children. These findings suggest that merozoite-specific IgM is an important functional and long-lived antibody response targeting blood-stage malaria parasites that contributes to malaria immunity.

## Teaser

IgM is an important and long-lived component of anti-malarial immunity in humans and blocks infection of red blood cells.

## INTRODUCTION

With the stagnation of reduction in malaria over the past 3 to 5 years despite ongoing global control efforts (*1*), development of new tools and effective vaccines for malaria control is strongly needed. The most advanced malaria vaccine, RTS,S, based on the pre-erythrocytic sporozoite stage, had only 26 to 36% efficacy in infants and young children receiving a booster dose (*2*). An important target of vaccine development is the blood stage of *Plasmodium falciparum*, where vaccines aim to prevent merozoite invasion of red blood cells (RBCs) (*3*). Antibodies against merozoites act to suppress parasite blood-stage replication and disease through multiple mechanisms, including direct inhibition of erythrocyte invasion, interactions with complement to lyse merozoites and inhibit invasion, and interactions with Fcγ receptors to promote phagocytosis (*3*). The overwhelming majority of research on protective human antibodies that target the merozoite has focused on the role of immunoglobulin G (IgG). In contrast, the potential importance of IgM in protective immunity has received comparatively limited attention, and much less is known about the dynamics of IgM acquisition and maintenance in malaria or how IgM might mediate protective effects.

The general view of IgM responses is that IgM is induced early by infection but then rapidly decays and is “replaced” by IgG after several weeks and in memory phase immunity. This paradigm of early IgM induction followed by conversion to IgG has been demonstrated in human responses to multiple infections including dengue (*4*), West Nile virus (*5*), and HIV (*6*), among others. However, this dynamic has not been established for *P. falciparum* malaria in humans, and relatively abundant levels of malaria-specific IgM have been reported in some studies of populations with high malaria exposure (*7*–*9*). Furthermore, the existence of *P. falciparum–*specific IgM memory B cells was recently demonstrated in samples from naturally exposed human subjects and mouse models of malaria (*10*). We hypothesized that the induction and maintenance of IgM responses to malaria may not follow the typical profile seen in other diseases and animal models and that IgM may have an important role in immunity to *P. falciparum* not only in primary infection but also during subsequent infections.

Here, we define the acquisition, maintenance, and decay of merozoite-specific IgM during *P. falciparum* infection and clinical malaria in humans in clinical trials of experimental blood-stage malaria infection in naïve individuals and among cohorts of naturally exposed individuals with differing levels of malaria exposure, including children and adults. We provide evidence that specific IgM not only is rapidly induced following a primary *P. falciparum* infection in malaria-naïve adults but also is prominent in most patients during malaria disease and *P. falciparum* infection in both children and adults with lifetime malaria exposures in endemic areas of Sabah, Malaysian Borneo, and Papua New Guinea (PNG). Further, IgM was a long-lived response in the absence of reinfection. To provide a mechanistic link to immunity, we demonstrate that IgM can inhibit merozoite invasion of RBCs and blood-stage replication by the fixation and activation of complement on the surface of merozoites. IgM to the merozoite surface increased with age and was associated with protection in a longitudinal cohort of naturally exposed children. Our data suggest that IgM responses, alongside IgG, are important contributors to naturally-acquired immunity against malaria.

## RESULTS

### Merozoite-specific IgM is rapidly induced during a low-dose primary *P. falciparum* infection

**Fig. 1:**
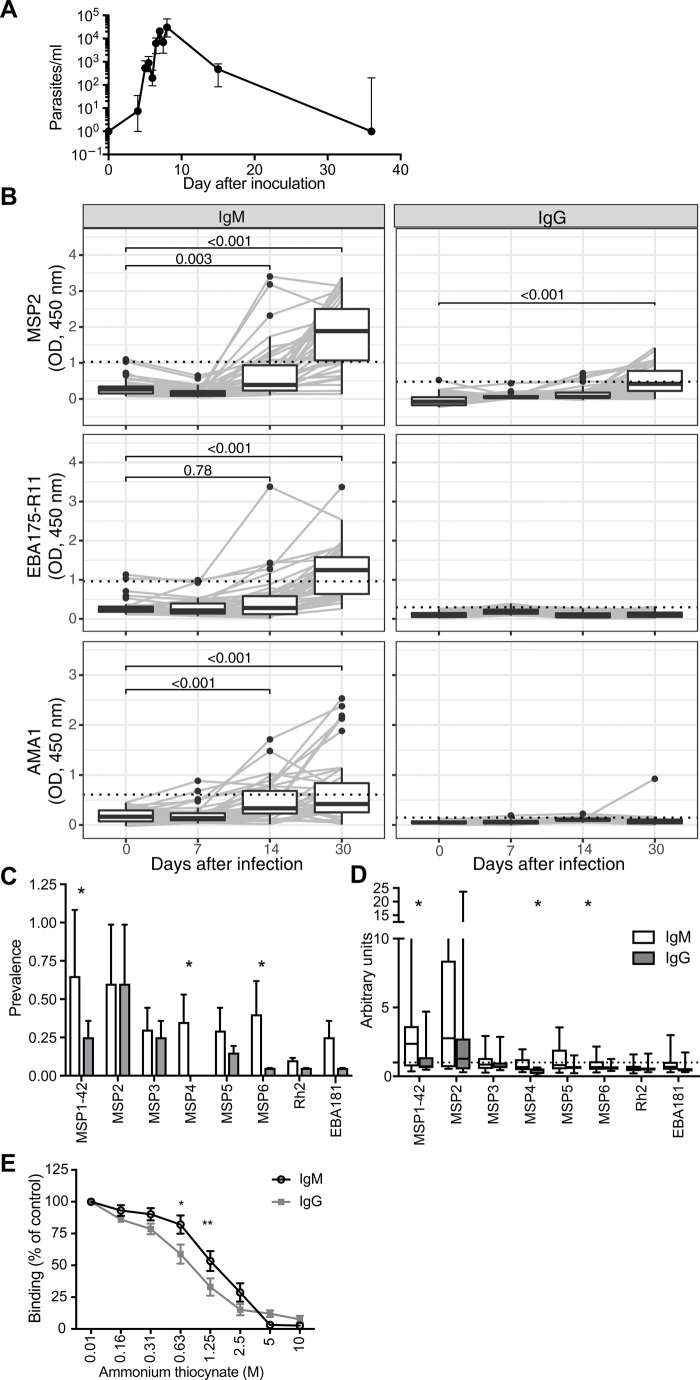
Induction of IgM and IgG following controlled human *P. falciparum* infection. (**A**) Malaria-naïve healthy individuals underwent inoculation with 1800 or 2800 viable *P. falciparum* 3D7-parasitized RBCs, and peripheral parasitemia was measured by qPCR. Participants were treated with anti-malarial drugs at day 7 or 8 of infection. Blood samples from 60 volunteers (from 10 independent cohorts) were collected before infection (day 0), at peak infection (day 7 or 8), and 14 or 15 and 28 to 36 days after inoculation; in analysis, these time points are grouped as days 0, 7, 14, and 30. Data are representative of 10 individuals from four cohorts. (**B**) IgM and IgG levels were measured against MSP2, AMA1, and EBA175 (RII) at days 0, 7, 14, and 30 following controlled human *P. falciparum* infection in malaria-naïve adults (*n* = 40). Antibody responses are expressed as optical density (OD) after background subtraction of no-serum wells. Positivity threshold was calculated as means + 3SDs of responses at day 0 (dotted line). *P* values are Wilcoxon matched-pairs signed-rank test. (**C**) IgM and IgG responses to several merozoite antigens were measured at day 30 following infection in CHMI participants (*n* = 20). Prevalence of antibody responses was considered as a positive response if antibody levels were greater than means + 3SDs of responses at day 0. Data are proportion positive, and error bars are upper 95% confidence interval calculated by the Wald method. *P* value is calculated by Fisher’s exact test. (**D**) Magnitude of responses relative to day 0 was assessed as arbitrary units (AU = OD day 30/means + 3SDs of responses at day 0). *P* values are Wilcoxon matched-pairs signed-rank test. (**E**) Affinity of IgM and IgG to MSP2 was assessed by the dissociation of antibodies using increasing concentrations of ammonium thiocynate. Data are means ± SEM of eight individuals tested in two assays in duplicate, expressed as the percentage of OD in PBS. *P* values indicate Sidak’s multiple comparison test of IgM verses IgG from two-way analysis of variance (ANOVA), taking into account repeat measures for each individual (*P < 0.05 and **P < 0.01).

To investigate the induction of specific IgM in a first malaria infection, we assessed the kinetics of malaria-specific antibodies acquired in response to blood-stage controlled human malaria infection (CHMI) in naïve Australian adults (*11*). Subjects were infected with blood-stage *P. falciparum* and treated when blood parasite levels reached 20,000 (1050 to 107,980) parasites/ml [median (minimum to maximum); Fig. 1A]. In this experimental human model, we recently showed that IgM against a number of merozoite surface antigens is detectable a month after parasite infection (*12*). Here, we measured IgM and IgG levels in serum samples collected from participants to three merozoite vaccine candidates (MSP2, AMA1, and EBA175) at days 7 (day of treatment), 14, and 30 following *P. falciparum* inoculation and standardized responses compared to pooled immune adult PNG sera. While there was limited background reactivity of IgM before inoculation (day 0), there was clear evidence for the induction of antigen-specific IgM between 14 and 30 days after inoculation, indicated by large increases in antibody reactivity (Fig. 1B). There was no evidence for IgM or IgG induction at day 7 (day of treatment), indicating that induced antibodies likely had little role in parasite clearance following drug treatment. To confirm that increased IgM was due to a specific IgM response and not due to a global increase in nonimmune IgM, we tested for IgM reactivity to a nonmalarial protein before and following CHMI; there was no evidence for increased nonimmune IgM reactivity (fig. S1A). There was also a significant induction of IgG to MSP2 by 30 days. There was a significantly higher proportion of positive IgM responses compared to IgG responses by day 30 (table S1). To assess the relative breadth of IgM and IgG following controlled human *P. falciparum* infection, antibodies were quantified against a panel of merozoite surface proteins at 30 days following parasite inoculation in a second group of volunteers. Overall, there was a significant detection of IgM against all eight antigens tested (MSP1, MSP2, MSP3, MSP4, MSP5, MSP6, PfRh2, and EBA181) but only a significant detection of IgG to four of these antigens (MSP1, MSP2, MSP3, and MSP5; fig. S1, B and C). There was a higher prevalence of seropositive of IgM responses to MSP1, MSP4, and MSP6 compared to IgG (Fig. 1C). Similarly, the magnitude of antibody induction above background levels (arbitrary units) appeared greater for IgM than IgG for MSP1, MSP4, and MSP6 but not other antigens (Fig. 1D). However, caution should be taken when comparing magnitudes of responses for IgG versus IgM because of possible differences in sensitivity of secondary detection antibodies. We explored the avidity of IgM and IgG to MSP2 among a subset of samples and found that avidity was higher for IgM than for IgG (Fig. 1E). We studied antibodies to MSP2 because this was a prominent response observed in CHMI (Fig. 1B**)**.

### Merozoite-specific IgM appears prominent in clinical malaria and has similar kinetics to IgG following infection

**Fig. 2:**
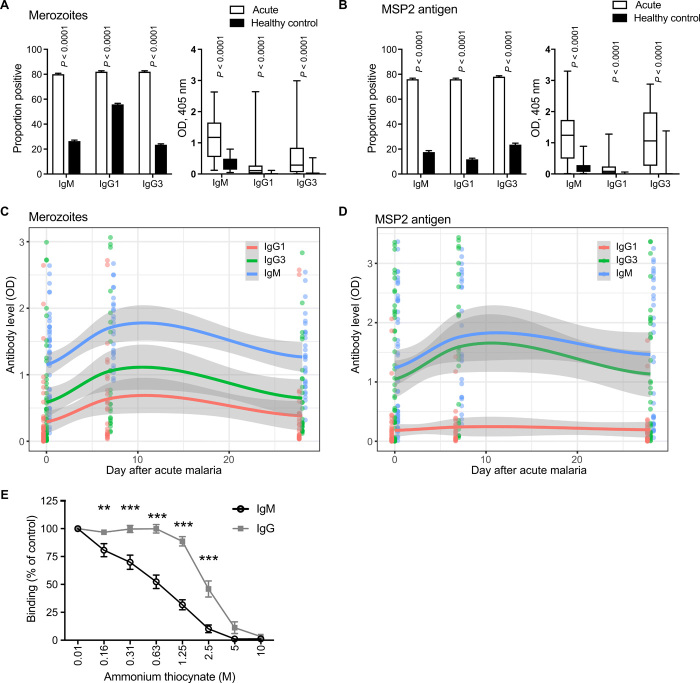
Induction and waning kinetics of merozoite-specific IgM during naturally acquired *P. falciparum* malaria. (**A** and **B**) IgM, IgG1, and IgG3 binding to intact merozoites (A) and recombinant MSP2 (B) was assessed among individuals with clinical malaria (*n* = 50) and uninfected endemic community controls (*n* = 30) in individuals in Sabah, Malaysian Borneo. Antibody reactivity is shown as seroprevalence and media [plus interquartile range (+IQR)] OD. Seroprevalence (left) was calculated using a cutoff defined as the mean OD + 3SDs of unexposed Australian naïve controls. (**C** and **D**) IgM, IgG1, and IgG3 binding to intact merozoites (C) and recombinant MSP2 (D) was assessed in patients with clinical malaria in Sabah, Malaysian Borneo, and at 7 (*n* = 26) and 28 days (*n* = 32) after anti-malarial treatment. Solid lines and gray area are generated from LOWESS smooth calculations in R software. Raw data are indicated as points. (**E**) Affinity of IgM and IgG to MSP2 was assessed by the dissociation of antibodies using increasing concentrations of ammonium thiocynate. Data are means ± SEM of eight individuals tested in two assays in duplicate, expressed as the percentage of OD in PBS. *P* values indicate Sidak’s multiple comparison test of IgM versus IgG from two-way ANOVA, taking into account repeat measures for each individual (**P < 0.01 and ***P < 0.001).

We next quantified the induction and waning of IgM during a malaria clinical episode in children and adults in a malaria-endemic region of Sabah, Malaysian Borneo, who had a lifetime exposure to malaria infection (table S2). We tested serum samples for specific IgM to intact merozoites and recombinant MSP2. We also measured cytophilic IgG subclass responses, IgG1 and IgG3, that are associated with protection from malaria (*3*). During clinical malaria, a higher proportion of patients was IgM seropositive and had a higher magnitude of IgM compared to uninfected healthy community controls for merozoites and MSP2 [merozoites: prevalence, 80% versus 26% (*P* < 0.0001) and magnitude median optical density (OD), 1.17 versus 0.2 (*P* < 0.0001); MSP2: prevalence, 76% versus 18% (*P* < 0.0001) and magnitude median OD, 1.24 versus 0.11 (*P* < 0.0001) Fig. 2, A and B]. There was no difference in IgM seropositivity (80%) compared to IgG1 (82%) or IgG3 (82%), suggesting that, by the time symptomatic disease develops, both IgM and IgG responses have been established in populations with prior malaria exposure (Fig. 2, A and B**)**. In a subset of patients, sera were available at days 7 and/or 28 following treatment. Among these, there was a significant increase in IgM levels to intact merozoites and MSP2 at 7 days following treatment, which reduced to levels seen during clinical malaria by 28 days following treatment. There was no apparent difference in the pattern of induction and decline of IgM compared to IgG1 and IgG3 subclasses (Fig. 2, C and D, and fig. S2, A and B). When patients were divided into children (<15 years old) and adults, reflecting different levels of cumulative malaria exposure, there was no difference in rates of seroprevalance of IgM or IgG subclasses to intact merozoites. For MSP2 responses, there was a significantly higher prevalence of IgM and IgG1, but lower prevalence of IgG3, in children compared to adults [89% versus 68% (*P* < 0.0001) for IgM and IgG1, respectively, and 73% versus 81% (*P* = 0.006) for IgG3; fig. S2C]. Children also had slightly higher magnitude of IgM to the merozoite surface (*P* = 0.04) but not to MSP2 during clinical malaria (*P* = 0.63; fig. S2, D and E**)**. The higher IgM in children was not apparent following treatment (fig. S2, D and E). In avidity assays among a subset of samples, the avidity of IgG was higher than that of IgM (Fig. 2E) and appeared higher than the IgG avidity observed in the CHMI studies.

### Merozoite-specific IgM is maintained for at least 12 months following *P. falciparum* malaria

**Fig. 3:**
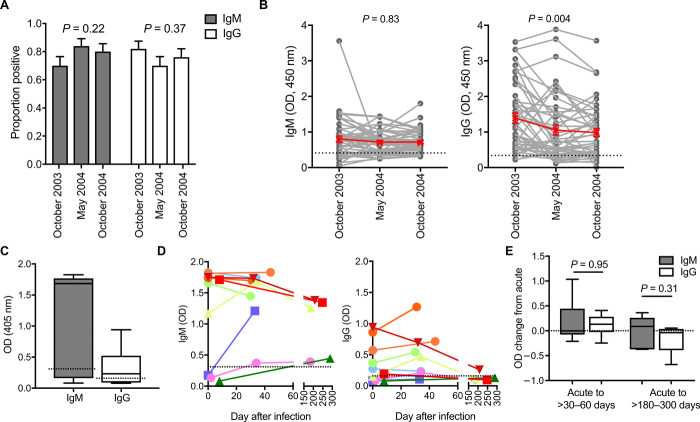
Maintenance of *P. falciparum* specific IgM and IgG. (**A** and **B**) IgM and IgG binding to recombinant MSP2 was measured in children from Ngerenya, Kenya who had no detectable parasite infection in October 2003, May 2004, and October 2004, during a period of rapidly declining transmission (*n* = 50). (A) IgM and IgG prevalence at the three time points (seropositive defined as greater than means + 3SDs of reactivity of malaria-naïve donors). *P* is Chi-square test. (B) Magnitudes of IgM and IgG reactivity over three time points. *P* value is calculated by nonparametric ANOVA and Friedman test. Dotted line indicates positive threshold calculated from means + 3SDs of reactivity of malaria-naïve donors. (**C**) IgM and IgG binding to recombinant MSP2 during clinical malaria in Australian returned travelers (*n* = 10). Dotted line indicates the seropositive threshold. (**D**) Magnitudes of IgM and IgG were measured at different time points following acute illness and treatment (*n* = 10). Colored lines correspond to different individuals. Dotted line indicates seropositive threshold. (**E**) There was no significant difference in the change in IgG and IgM levels at 30 to 60 days (*n* = 8) or between 180 to 300 days (*n* = 5) after disease compared to clinical time point (measured 0 to 7 days following treatment). Dotted line indicates no change. *P* values were calculated using the Wilcoxon matched-pairs signed-rank test.

To quantify IgM kinetics over a longer duration after infection, we assessed IgM and IgG levels in serum samples collected from individuals living in a malaria-endemic region of Kenya (Ngerenya, Kilifi District) at three time points during a period of rapidly declining malaria transmission intensity (*13*). We examined IgM and IgG antibodies to MSP2 among a subset of children who had no recorded episode of parasitemia between October 2003 and October 2004. We found that 70% of children had detectable IgM to MSP2 in October 2003, and prevalence was not significantly different to IgG (IgG prevalence, 82%; *P* = 0.16). IgM and IgG responses were weakly correlated (rho = 0.386, *P* = 0.006). There was no change in IgM or IgG prevalence rates between October 2003 and October 2004 (Fig. 3A). There was no overall change in the magnitude of IgM responses over time (*P* = 0.83), but there was a significant decline in IgG magnitudes between October 2003 and May 2004 (*P* = 0.004; Fig. 3B).

Longevity of IgM was also observed in serum samples in returned travelers who were residents of Australia, with no prior malaria episodes, who acquired malaria while traveling; in this cohort, we could exclude any potential reinfection that could influence antibody kinetics during follow-up (table S3). At the time of clinical malaria, both IgG and IgM were detected (Fig. 3C). In the year following clinical malaria, there was no apparent difference in IgG and IgM decay within individuals. Even 6 months after malaria, detectable levels of IgM but not IgG were still present in all returned travelers’ serum samples tested (Fig. 3D). To compare the overall loss of antibodies, the change in antibody levels was calculated at two time points following clinical malaria, between 30 and 60 days or between 180 and 300 days. There was no difference in the change in IgM compared to IgG at either time points, indicating that IgM was not more rapidly lost than IgG following malaria (Fig. 3E).

### IgM inhibits merozoite invasion via complement fixation and activation

**Fig. 4:**
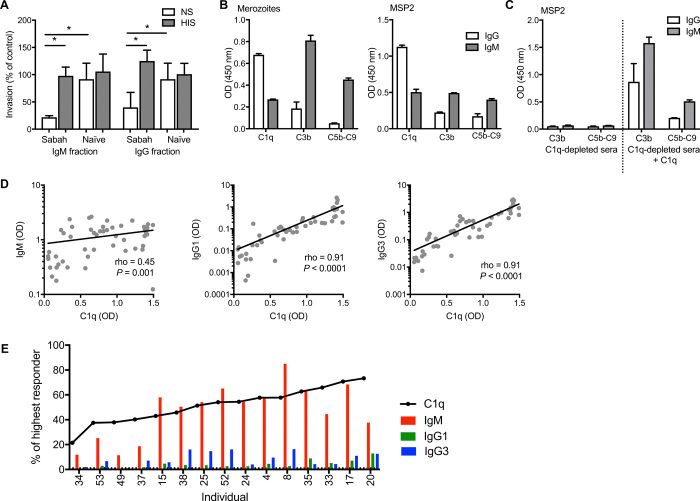
Merozoite-specific IgM inhibits invasion in a complement-dependent manner. (**A**) Isolated IgM and IgG fractions at 50 μg/ml from malaria-exposed donors (Sabah, Malaysian Borneo) or naïve donors (Australia) were tested in merozoite invasion inhibition assays (at 50 μg/ml) with normal serum (NS) or heat-inactivated serum (HIS) from malaria-naïve donors as a source of complement. Data are expressed as the percentage of invasion in heat-inactivated serum, with no antibody control. Data are means ± SD of two assays performed in duplicate. Significant differences are indicated as *P* < 0.05 by Mann-Whitney *U* test. *P < 0.05. (**B**) Isolated IgM and IgG fractions were tested for deposition of complement components (C1q, C3b, and C5b-C9) on intact merozoites or recombinant MSP2 by ELISA with 10% normal sera from malaria-naïve donors as a source of complement. Data represent the level of complement binding measured in OD values minus deposition observed using naïve IgM and IgG. (**C**) IgM and IgG fractions were tested for deposition of complement components on recombinant MSP2 by ELISA with 10% C1q-depleted sera and C1q-depleted sera reconstituted with purified C1q (10 μg/ml). Data are means ± SEM of two independent assays performed in duplicate. (**D**) The level of C1q-fixing antibodies and IgM and IgG1/IgG3 isotypes to merozoites was quantified in 50 individuals with a clinical malaria episode from Sabah. IgM, IgG1, and IgG3 were correlated with C1q fixation. (**E**) To compare directly between C1q fixation and IgM or IgG1/IgG3, antibody levels were converted to arbitrary units (the percentage of the highest measured response within cohort) and ranked according to levels of C1q fixation. In some individuals with relatively high C1q-fixing antibodies (defined as >40% of highest responder), IgM dominated the antibody response targeting the merozoite.

We recently demonstrated that a key functional mechanism of merozoite-specific IgG is complement fixation (*14*). We found that IgG interacted with the first component of the classical complement cascade, C1q, to directly inhibit RBC invasion (in the absence of other complement components) and to fix complement on the merozoite surface, leading to lysis (*14*). IgM is also known to bind C1q and activate the classical complement pathway. To assess the capacity of IgM to target merozoites via complement fixation, we separated IgM and IgG fractions from serum pooled from patients within our Sabah cohort using protein G columns. Bound fractions contained IgG, while unbound IgG-depleted fractions contained IgM and are referred herein as “IgM fractions” for clarity of reading. The purity of IgG and IgM fractions was assessed via enzyme-linked immunosorbent assay (ELISA) and Western blot. There was minimal IgM in the IgG fraction and no remaining IgG detectable in the IgM fraction (fig. S3, A to C). IgM and IgG fractions at 50 μg/ml were tested in established invasion inhibition assays with isolated merozoites (*15*). Both IgM and IgG fractions from malaria-exposed individuals effectively inhibited merozoite invasion in the presence of normal nonimmune serum (complement active) (Fig. 4A). There was no evidence that IgM or IgG fractions directly inhibited merozoite invasion in the absence of active complement, as invasion was comparable to naïve antibody samples when tested in heat-inactivated serum (complement inactive) (Fig. 4A).

To further support the role of complement in IgM inhibitory activity, we demonstrated that IgM could fix and activate complement on intact merozoites and MSP2. Levels of C1q (the primary component of the classical complement cascade), and downstream components C3b and C5b-C9 (which form the membrane attack complex in the cell surface to mediate lysis), were quantified. Both IgM and IgG fractions effectively fixed C1q, leading to the formation of C3b and C5b-C9 on the surface of the merozoite and on recombinant MSP2 (Fig. 4B). On intact merozoites, the level of C1q fixation was twofold higher with IgG compared to IgM. However, it was notable that the level of C3b and C5b-C9 deposition was ninefold higher with IgM compared to IgG fractions; relative to the level of C1q fixation, C5b-C9 formation was ~20-fold higher for IgM. Similar patterns of C1q, C3b, and C5b-C9 deposition were seen with recombinant MSP2 (Fig. 4B). This suggests that IgM is substantially more effective at activating the complement cascade to promote C5b-C9 formation. This high activity is despite lower avidity of IgM compared to IgG (fig. S3D). Supporting the high complement-fixing capacity of IgM, we observed that IgM and complement promoted merozoite lysis more efficiently than IgG, reflecting the formation of membrane attack complex and its effects on merozoites (fig. S4). To confirm that complement activation was occurring via the classical complement cascade, complement activation on MSP2 by IgM and IgG was assessed using C1q-depleted sera. There was minimal formation of complement components C3b and C5b-C9 for both IgG and IgM fractions using C1q-depleted sera. Deposition of C3b and C5b-C9 was regained with the reconstitution of depleted sera with C1q (Fig. 4C).

### Relationship between complement fixation, IgM, and IgG

We have recently shown that the capacity of antibodies to fix complement to specific merozoite antigens is strongly associated with protection (*16*). To investigate the relative importance in IgM in complement-dependent immunity targeting the merozoite, we assessed the relationship between IgM, IgG1, and IgG3 subclass antibodies and C1q-fixing antibody activity to purified merozoites using serum samples from patients with a clinical malaria episode in Sabah. Magnitudes of C1q-fixing antibodies were tightly correlated with formation of downstream complement components C3b and C5b-C9 (fig. S5A) (*16*). Similar to antibody responses (Fig. 2A), the prevalence and magnitude of C1q-fixing antibodies were higher in participants with clinical malaria compared to healthy endemic controls [prevalence, 73% versus 6% (*P* < 0.0001); median OD, 0.94 versus 0.2 (*P* < 0.0001); fig. S5, B and C]. The proportion or magnitude of C1q-fixing antibodies was not different between adults and children during clinical malaria (fig. S5, D and E). Levels of C1q-fixing antibodies significantly correlated with IgM responses (rho = 0.45, *P* = 0.001). There was also a strong correlation between IgG1 and IgG3 with C1q, reflecting a substantial contribution of these IgG subclass in mediating C1q fixation (for both IgG1 and IgG3, rho = 0.91, *P* < 0.0001; Fig. 4D). However, 30% of individuals (15 of 50) were identified with relatively high C1q-fixing antibodies but relatively low levels of IgG1 and IgG3 (Fig. 4E), suggesting that, in the absence of IgG complement-fixing subclasses, IgM responses were important in mediating C1q fixation to *P. falciparum*.

### Merozoite-specific IgM is associated with protection from malaria in naturally exposed children

**Fig. 5:**
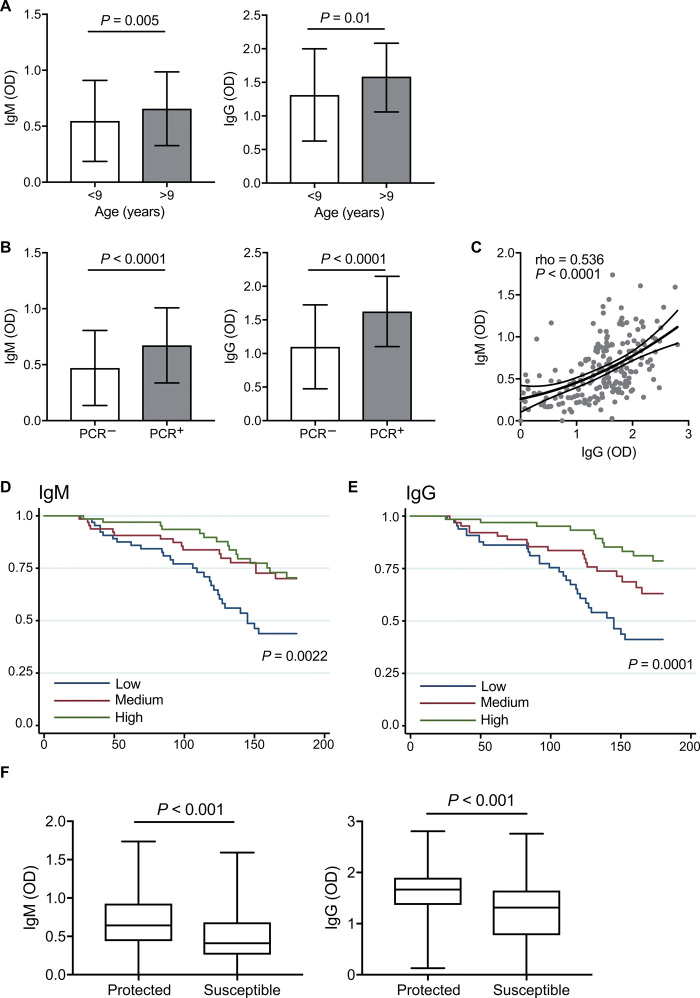
Association between IgM and IgG levels to the merozoite and malaria risk. IgM and IgG levels to the merozoite surface were measured in plasma from a cohort of 199 children in PNG. (**A**) IgM and IgG were higher in older children (>9 years, *n* = 109). (**B**) High IgM and IgG binding were higher in children with concurrent *P. falciparum* infection (*n* = 134) than in uninfected children, as determined by PCR. (**C**) Correlation between IgM and IgG among children (Spearman rho and *P*) is indicated. (**D** to **G**) Children were stratified into three equal-sized groups based on tertiles according to low (including those classified as “negative”), medium, or high IgM and IgG responders, as determined by OD values for each sample. (**D** and **E**) High IgM and IgG binding to the merozoite was strongly associated with reduced risk of clinical malaria episodes. (**F**) Children who had at least one infection during the follow-up period (*n* = 190) were allocated into group defined as protected (always asymptomatic; *n* = 112) or susceptible (experiencing at least one malaria episode; *n* = 78) in follow up. *P* values calculated by Wilcoxon rank sum between groups are indicated.

To investigate the potential importance of IgM alongside IgG in protection from malaria, we measured IgM and IgG levels in plasma samples against intact merozoites in 199 children (5 to 14 years old) from a longitudinal cohort in a moderate-to-high malaria transmission region of PNG (*17*); all children were treated to clear parasitemia at enrolment and then monitored by active surveillance for parasitemia and clinical malaria over 6 months of follow-up. The prevalence of IgM and IgG reactivity to the merozoite surface was very high at enrolment (90 and 99.5%, respectively; table S4). Both IgM and IgG levels were associated with age, such that older children had significantly higher IgM and IgG levels than younger children, consistent with acquisition of both antibody isotypes with increased cumulative exposure among older children (Fig. 5A and table S4). Both IgM and IgG were also higher in children who were parasitemic at the time of sample collection compared to uninfected children, suggesting that both antibody isotypes are boosted during infection (Fig. 5B). IgM and IgG levels were moderately positively correlated, suggesting co-acquisition of isotypes to some extent (Spearman’s rho = 0.534, *P* < 0.001; Fig. 5C).

Children with high levels of IgM at enrolment were associated with a 53% [95% confidence interval (CI), 0.25 to 0.89] reduction in the risk of clinical malaria (fever plus >5000 parasites/μl) compared to children with low levels of IgM after adjusting for confounders (age and location of residence) (Table 1 and Fig. 5D). For comparison, children with high levels of IgG at enrolment were associated with a 70% (95% CI, 0.14 to 0.64) reduction in the risk of clinical malaria (Table 1 and Fig. 5E). The magnitude of the risk reduction associated with high IgM and high IgG was less than seen for the previously reported protective effect of complement-fixing activity of antibodies within the same children [85% reduced risk of clinical malaria (*14*)]. This suggests that it is the functional capacity of antibodies to fix complement that is most strongly linked with protection, rather than the magnitude of specific antibody isotypes, and complement fixation can be mediated by IgG and IgM.

**Table 1:** Association between IgM and IgG to the merozoite surface and risk of clinical malaria. The cohort (*n* = 199) was stratified into three equal groups according to low, medium, or high levels (defined by tertiles) of IgM to the merozoite surface. Unadjusted hazard ratios (uHRs) and HRs adjusted for the predetermined confounders of age and location of residence [adjusted HRs (aHRs)] are presented. IgM levels were compared between low-versus-medium (LvM) and low-versus-high (LvH) groups for the risk of symptomatic malaria (fever plus >5000 parasites/μl). Calculations were based on the first episode only.

		**uHR [95% CI]**	***P***	**aHR [95% CI]** **age/location**	***P***
**IgM**	LvM	0.44 [0.23–0.81]	0.009	0.62 [0.32–1.18]	0.15
	LvH	0.38 [0.20–0.81]	0.002	0.47 [0.25–0.89]	0.02
**IgG**	LvM	0.51 [0.29–0.91]	0.021	0.58 [0.32–1.06]	0.08
	LvH	0.24 [0.12–0.49]	<0.001	0.3 [0.14–0.64]	0.002

In complementary analysis, children who had at least one recorded infection in follow up (*N* = 190) were classified as either protected from clinical malaria (always asymptomatic when infected) or susceptible to clinical malaria (experiencing one or more clinical malaria episodes); children with no recorded infections were excluded to ensure that only children who were exposed were included in the analysis. Children who were protected had significantly higher levels of IgM than susceptible children (Fig. 5F). IgG levels were also higher in children who were protected. These associations remained significant in logistic regression models (adjusted for age and location of residence); high levels of IgM were associated with 73% reduced odds of susceptibility, and IgG was associated with 77% reduced odds of susceptibility. These protective associations were somewhat lower than that observed for high C1q-fixing antibodies, as measured previously (*14*), which were associated with an 89% reduced odds of susceptibility (table S5), further supporting a concept that functional activity is the main determinant of protection, and this activity is mediated by both IgG and IgM.

## DISCUSSION

Antibodies that target the *P. falciparum* merozoite and prevent blood-stage infection are key mediators of protective immunity. However, studies have largely focused on understanding IgG responses. The dynamics of IgM induction and maintenance and its functional activity that could contribute to protection have not been established. Here, we show that IgM was rapidly induced during a primary low-dose *P. falciparum* infection of malaria-naïve adults and appeared to be prominent in most patients with clinical malaria in individuals with life-long malaria exposure, including adults. Contrary to the expectation that IgM is a short-lived response to primary infection, we found that IgM responses persisted after infection with no apparent difference in antibody decay rates between IgM and IgG in Kenyan children or following a primary malaria infection acquired in Australian travelers. Providing a mechanistic link between IgM and immunity, we found that IgM inhibited merozoite invasion in a complement-dependent manner and demonstrated the fixation of complement to the merozoite surface by IgM. Notably, IgM was markedly more effective at activation of complement on the merozoite surface than IgG. Consistent with a potential role for IgM in contributing to protective immunity, merozoite-specific IgM increased with age and was associated with reduced risk of clinical malaria in a longitudinal cohort study. Overall, our data provide substantive new evidence supporting IgM as an important functional antibody that targets merozoites and may be a significant contributor to naturally acquired protective immunity to malaria. We demonstrate that IgM is a substantial component of antibody responses to malaria even among those with extensive prior malaria exposure and that IgM persists following acute infection. This suggests a rethink of the existing paradigm of early IgM induction followed by replacement of the IgM response with IgG.

We showed that the kinetics of acquisition and decay of naturally acquired merozoite-specific IgM and IgG were not apparently different in multiple cohorts. This is despite there being differences in the estimated circulating half-lives of each antibody type, suggesting that, during acute infection and convalescence, detected antibodies result from continued production by short- or long-lived antibody-secreting cells [approximate half-lives, 36 days (IgG1) and 28 days (IgG3) (*18*) compared to 5 days (IgM) (*19*)]. Long-term maintenance of IgM was detected in children living in Kenya during a period of low malaria transmission in the absence of detected infection. Magnitudes of IgM showed no changes within these children, while there was evidence of declining IgG. Supporting these findings, in travelers returning to Australia from malaria-endemic areas, IgM was detected in all individuals longer than 6 months after malaria infection. Studying these subjects allowed us to exclude the possible boosting or re-emergence of IgM due to reinfection. Together, results suggest that there is induction and maintenance of long-lived IgM-secreting cells and/or memory IgM^+^ B cells following clinical malaria.

Consistent with these findings, *Plasmodium-*specific somatically hypermutated memory IgM^+^ B cells were recently detected in mouse models of malaria and in humans from regions of natural malaria transmission (*10*). Further, using immune repertoire sequencing, it was demonstrated that IgM memory B cells can maintain IgM expression after an acute malaria episode in young Malian infants (*20*), although the kinetics and functions of IgM were not defined. In a mouse model using a rodent *Plasmodium* species, somatically hypermutated memory IgM^+^ B cells were essential in the generation of both IgM- and IgG-secreting plasmablasts during secondary infection (*10*). This finding indicates that IgM responses may have essential roles not only directly, due to IgM secretion, but also in maintaining and boosting IgG responses. However, the concepts of sustained maintenance of circulating IgM antibodies in human immunity, and its presence even among those with extensive prior malaria exposure, have not been previously established. While we have indirectly shown that IgM antibodies measured here are from memory B cells, our data are suggestive of IgM memory acquisition; in our cohort of PNG children, from an area of relatively high malaria transmission intensity, older children who have had greater overall malaria exposure had both higher merozoite-specific IgM and IgG levels compared to younger children, consistent with the acquisition of both memory antibody isotypes with increasing immunity. Thus, in contrast to other pathogens (*4*, *5*, *21*), anti-malaria IgM not only is a feature of primary infection that is subsequently replaced by IgG but also increases as immunity is acquired. Recent findings have also shown that IgM increases with age against the important sporozoite antigen and vaccine target CSP (circumsporozoite protein) (*22*). Further studies are required to specifically investigate the induction of IgM memory responses and how they contribute to the increased magnitudes of parasite-specific IgM observed with increasing age and parasite exposure. Detailed studies of IgM responses before and after repeat malaria episodes in longitudinal cohort studies may also help determine the significance of IgM memory. Further studies on the roles of IgM responses against multiple stages of the *P. falciparum* life cycle are also warranted, especially because antibody complement fixation appears to be important for immunity against sporozoites (*22*) and gametes (*23*).

Merozoite-specific IgM blocked merozoite invasion of RBCs in a complement-dependent mechanism, which has been previously reported only for IgG (*14*). Complement activation was via the classical complement cascade, with the first component of the classical cascade C1q, along with downstream components C3 and C5b-C9 detected on both intact merozoites and recombinant MSP2; complement fixation by antibodies was absent when using C1q-depleted sera rather than normal serum. The capacity of IgM to activate complement leading to C5b-C9 and merozoite lysis was substantially higher than IgG at similar concentrations; IgM led to much higher C5b-C9 formation relative to the fixation of C1q. Among these malaria-exposed samples, IgM had a relatively lower avidity than IgG in tested samples but still had similar or superior complement-activating activity. This suggests that the acquired IgM and IgG have antibody affinities that are above a threshold required for complement activation. In related studies, we found that antibody affinity does not correlate with complement fixation activity, probably because antibody avidities are well above the threshold required to fix and activate complement (*16*). IgM has been estimated to trigger a complement response that is many-fold more effective than IgG (*24*). This greater activity of IgM further highlights the potential importance of IgM in immunity and the need for further studies to investigate IgM responses in malaria vaccine development. This is the first study to identify a direct functional mechanism of naturally acquired human IgM in targeting *P. falciparum* blood-stage replication. It has been also previously reported that IgM in the presence of mononuclear cells is able to prevent parasite growth in vitro (*25*). While IgM does not bind to classical FcR, IgM has been reported to mediate phagocytosis via Fcα/μR (*26*) and complement receptor 3 (*27*), and the IgM receptor FcμR has been reported to be expressed on human activated macrophages and circulating monocytes (*28*), suggesting additional roles for IgM in immunity to malaria that should be investigated in future studies.

Supporting a potential role of IgM in contributing to parasite clearance and protection, we found that high levels of merozoite-specific IgM were associated with protection from clinical malaria in a cohort of naturally exposed children from PNG (*17*). This protective association was comparable to that observed for high IgG, but both IgM and IgG protective associations were lower than that previously reported for C1q-fixing antibodies in the same cohort (*14*). This might suggest that both IgG and IgM mediate complement fixation that contributes mechanistically to protection from malaria. IgM responses to some merozoite antigens have been also associated with protection in some longitudinal studies in humans (*7*–*9*), but IgM responses to whole merozoites have not been previously reported. Because of the difficulties in quantifying the magnitude of parasite-specific IgG and IgM in individuals, we are unable to directly assess the relative importance of each isotype in complement fixation in our cohorts. C1q-fixing antibodies were strongly associated with both IgM and IgG1 and IgG3 subclasses, and we are not able to assess which antibody isotype contributes more significantly to complement fixation and protection in vivo. However, IgM appeared to be important in mediating complement fixation in a proportion of individuals who had low levels of IgG1 and IgG3. Further, serum IgM preparations following the removal of IgG effectively fixed complement on merozoites and inhibited invasion in vitro. Together, results suggest that it may be the overall capacity of antibodies to mediate complement fixation to the parasite, rather than specific IgM or IgG isotypes that is required for protection. Further studies in additional populations will help define the relative importance of IgM, IgG, and complement fixation in immunity.

Avidity was higher for IgM than IgG in controlled human malaria infection studies where subjects experienced their first malaria infection, whereas IgG avidity was higher in residents of Sabah, reflecting affinity maturation from repeated malaria exposure and over time. This difference in avidity did not appear to be important for complement fixation, suggesting that affinity maturation of IgG responses may be important for other reasons, such as B cell selection or possibly Fcγ receptor interactions. It is possible that IgM affinity maturation also occurs with repeated exposure, but we were not able to directly assess this. However, our data do suggest that the extent of any affinity maturation in IgM is less than seen for IgG responses to malaria. In contrast to antigen-specific IgM responses studied here, a prior study reported that nonimmune IgM can bind to merozoite surface antigens DBLMSP1 and DBLMSP2 (*29*). In our studies, we found only low levels of reactivity of nonimmune IgM to merozoites compared to high reactivity of malaria-specific IgM to merozoites.

Currently, the functional roles and kinetics of IgM responses have been largely undefined and IgM has had limited research focus. This may partly be because IgM is regarded largely as a feature of primary infections based on studies with other pathogens; however, we showed that that is not that case in *P. falciparum* malaria. Furthermore, the role of antibody-complement interactions in immunity to merozoites and methods to study these have been only recently established, and our studies here demonstrate that this is an important mechanism of IgM responses. Therefore, these new insights and approaches provide avenues for future research into IgM in malaria and may facilitate an enhanced focus on this response. Our findings also prompt questions regarding why IgM is produced in response to malaria and why responses persist even in those with extensive malaria exposure. An early IgM response to primary infection may be important because it has high avidity, owing to its pentameric structure, and it is a potent activator of complement. Therefore, early generation of specific IgM that can fix and activate complement may be important until high-affinity IgG response evolves. We speculate that IgM responses may continue to be a feature of immune responses to subsequent malaria episodes because of its strong ability to activate complement, which appears to be an important contributor to immunity (*14*, *16*, *22*). The limitation of IgM is that it does not interact with Fcγ receptors, and therefore, the evolution of high-affinity IgG is also crucial given its multiple functional activities. The findings that both IgG and IgM are prominent in immune individuals in different populations and that both isotypes have functional activity suggest that a balance of IgG and IgM responses may be important for providing effective immunity and preventing disease pathogenesis.

Our findings suggest that the role IgM in human malarial immunity needs greater attention and investigation in future studies of naturally acquired immunity and vaccine trials. Because of the co-acquisition of both IgG and IgM in human immunity to malaria, it is difficult to quantify the relative contribution of each antibody isotype to protective immunity to malaria. Future large longitudinal cohorts with repeated measures of antibody acquisition may shed light on these outstanding questions, and animal models may also be helpful in understanding the in vivo roles of IgM, IgG, and complement. Mouse models of malaria are widely used in malaria vaccine research; however, mouse strains currently used in these models have very low complement activity compared to humans (*30*), which limits the use of these for assessment of IgM, IgG, and complement in immunity. In addition, many *P. falciparum* merozoite antigens that are important targets of human immunity are not present in rodent malaria species (*3*). Therefore, investment in the development of new animal models is needed.

In summary, we present epidemiological evidence to show that IgM is rapidly induced following infection experimentally or by natural exposure and is maintained to a level similar to IgG following treatment. We showed that merozoite-specific IgM is an important part of the immune response to malaria in children and adults. IgM functions by preventing merozoite invasion of RBCs in a complement-dependent manner and is a potent activator of complement on the merozoite surface. In a cohort of naturally exposed PNG children, IgM was associated with protection from clinical malaria. Collectively, these findings change our understanding of the role and kinetics of IgM in malaria immunity, suggesting an important role of anti-malarial IgM in humoral immunity that could be considered in vaccine design.

## MATERIALS AND METHODS

### Study cohorts

#### Controlled human malaria infection participants

For participants in controlled human malaria infection, inoculum preparation, volunteer recruitment, infection, monitoring, and treatment were performed as previously described (*31*). Briefly, healthy malaria-naïve individuals underwent induced blood-stage malaria inoculation with 1800 or 2800 viable *P. falciparum* 3D7-parasitized RBCs, and peripheral parasitemia was measured at least daily by quantitative polymerase chain reaction (PCR). Participants were treated with anti-malarial drugs at day 7 or 8 of infection. Blood samples from 60 volunteers (from 10 independent cohorts) were collected before infection (day 0), at peak infection (day 7 or 8), and 14 or 15 and 28 to 36 days after inoculation; in analysis, these time points are grouped as days 0, 7, 14, and 30. Plasma was collected from lithium heparin whole-blood samples according to standard procedures, snap frozen in dry ice, and stored at −70°C. All studies were registered with the Australian and New Zealand Clinical Trial Registration scheme. Participants were healthy males and females between 18 and 55 years with no prior exposures to malaria or residence in malaria-endemic regions. Two groups of participants were tested. The first group (inoculated with 2800 viable *P. falciparum* 3D7-parasitized RBCs; *n* = 40) were drawn from clinical trials nos. NCT02867059, NCT02783833, NCT02431637, and NCT02431650. The second group (inoculated with 1800 viable *P. falciparum* 3D7-parasitized RBCs; *n* = 20), tested only on days 0 and 30, was drawn from clinical trial nos. NCT02453581, ACTRN12613001040752, and ACTRN12613000565741.

#### Australian returned traveler

Serum samples were collected from travelers aged 19 to 56 who had returned to Australia from malaria-endemic regions and presented to hospital with malaria. All patients had no previous malaria episodes (self-reported). Sera were collected within a week of presentation and at one or two additional time points following treatment (30 to 90 days and > 90 days).

#### Ngerenya (Kilifi district, Kenya)

Sera samples were collected from venous blood from participants in a cohort study conducted in Ngerenya (Kilifi district, Kenya), where biannual malaria transmission occurs (May to July and November to December). This cohort composed of approximately 300 children aged 0.5 to 10 years who were followed for 3 years from May 2002 to October 2004. Venous blood was collected in May and October from each year. At each time point, the presence of *P. falciparum* parasitemia was assessed among all children by light microscopy of blood smears. Throughout the cohort period, active malaria case detection was performed weekly; children who were febrile (temperature of ≥37.5°C) or had a recent history of fever or illness had a blood smear performed. For the purpose of this study, we assessed antibody responses during the period of minimal malaria transmission, only in children (*n* = 50) who were sampled at all three time points that corresponded to the decline in malaria prevalence (October 2003, May 2004, and October 2004), who were aparasitemic at all time points and intervals, and who were previously positive for antibodies to merozoite antigens at October 2003 (*13*).

#### Malaysian clinical malaria studies

For patients with clinical *P. falciparum* malaria from Sabah, Malaysia, plasma samples were collected from participants in prospective comparative studies of *Plasmodium knowlesi*, *P. falciparum*, and *Plasmodium vivax* malaria, including at three district hospital sites (Kudat, Kota Marudu, and Pitas) (*18*) and a tertiary referral center (Queen Elizabeth Hospital, Kota Kinabalu) (*32*). In adults, this cohort has a high proportion of infected males across all species, possibly because of infection risk of forest workers (*33*). Patients who were positive for *Plasmodium* species confirmed by microscopy and PCR, who had a fever at the time of presentation or a history of fever in the preceding 24 hours, and who provided consent, were enrolled The ages of participants included in this analysis were 3 to 55 years. Individuals who had been living in the area in the preceding 3 weeks, who were negative for *Plasmodium* spp. by microscopy and PCR, and who had no history of fever in previous 48 hours were enrolled as endemic healthy controls. Plasma was collected from lithium-heparin collection tubes at the time of presentation and follow-up visits at days 7, 14, and 28 after anti-malarial drug treatment. For this study, all participants with a *P. falciparum* mono-infection (not mixed with other *Plasmodium* spp. by PCR), and available sera samples were analyzed.

#### PNG children longitudinal (Mugil) cohort

For the longitudinal study of PNG children, plasma samples were obtained at enrolment from a prospective treatment reinfection cohort of 206 children aged 5 to 14 years (median = 9.3) in Madang, PNG (*17*). For the purpose of this study, 200 children were tested on the basis of sample availability. Children were actively reviewed every 2 weeks for symptomatic illness and parasitemia and for passive case detection over a period of 6 months. A clinical episode of *P. falciparum* malaria was defined as a fever and *P. falciparum* parasitemia of >5000 parasites/μl.

### Purification of IgG and IgM fractions

Pooled plasma from individuals from Sabah who had a recent malaria infection (*n* = 20) or from Australian residents supplied from Australian Red Cross blood bank (*n* = 15) were used for to generate IgG and IgM serum fractions. Antibodies were purified from pooled plasma via ammonium sulfate precipitation. Plasma was diluted to 20% in 0.9% NaCl (w/v), and saturated ammonium sulfate solution was added to a final concentration of 50% and incubated on ice for 30 min. Precipitate was pelleted at 10,000*g* and washed once with 50% ammonium sulfate solution before being resuspended in phosphate-buffered saline (PBS). Samples were dialyzed in PBS in dialysis tubing of 14-kDa molecular weight cutoff.

IgG and IgM fractions were separated by the 5-ml NAb Protein G Spin Column (Thermo Fisher Scientific) as per the manufacturer’s instructions. Ammonium sulfate–precipitated antibodies were incubated with columns for 10 min to bind IgG. Unbound supernatants, depleted of IgG but containing IgM, were collected; this IgG-depleted fraction is referred to as “IgM fraction” for clarity of reading. IgG was eluted from column with elution buffer and then dialyzed in PBS in dialysis tubing. To improve IgG depletion, IgM fraction was passed over the NAb Protein G Spin Column a second time before use in assays. To assess purity, fractions were run by SDS–polyacrylamide gel electrophoresis 4 to 20% polyacrylamide gel (Bio-Rad) in reducing sample buffer along with ChromPure IgM and IgG standards. Gels were visualized with Coomassie staining.

### Quantification of IgG and IgM

Total concentrations of IgM and IgG in each fraction were quantified with ELISA against ChromPure human IgM and IgG standards (Jackson ImmunoResearch). Standards and IgM- and IgG-purified fractions were coated in serial dilutions in 96-well flat bottom MaxiSorp plates (Nunc) at 4°C overnight in coating buffer (0.1 M sodium carbonate and 0.1 M sodium bicarbonate). Plates were washed in PBS-Tween 0.05% three times and blocked with 1% casein in PBS for 2 hours at 37°C, and IgG and IgM were detected with goat anti-human IgG horseradish peroxidase (HRP; Merck Millipore) and sheep anti-human IgM HRP (Chemicon Australia). Binding was detected with TMB (3, 3´,5,5´-tetramethylbenzidine) substrate (Sigma-Aldrich), and reaction was stopped using 1 M HCl. The optical density was read at 450 nm.

### *P. falciparum* parasite culture and merozoite invasion and lysis assays

*P. falciparum* D10-PfGPF isolates (*34*) were cultured in culture media of RPMI-Hepes (pH 7.4) supplemented with hypozanthine (50 μg/ml), gentamicin (20 μg/ml), 25 nM sodium bicarbonate, and 0.5% AlbuMAX II (Gibco) or 0.5% human sera in O^+^ RBCs from malaria-naïve donors (Australian Red Cross blood bank). Cultures were incubated in candle jars at 37°C. Parasite cultures were synchronized with heparin (Sigma-Aldrich) and used for merozoite isolation and invasion assays as described (*14*). Briefly, late-stage trophozoite and schizont cultures were purified by a MACS separation system (Miltenyl Biotec) and incubated with E64 protease inhibitor at 10 μg/ml to prevent schizont rupture. Once merozoite development was complete, merozoites were purified via filtration with 1.2-μm syringe filter. Purified merozoites were incubated with uninfected RBCs and normal (complement active) or heat-inactivated sera (complement inactive) at 50% final concentration, as well as IgG or IgM fractions as described (*14*). Invasion occurred for 30 min before cultures were washed and returned to standard culture conditions and allowed to mature for 40 hours. Cultures were stained with hydroethidine (40 μg/ml; Molecular Probes) and then counted by flow cytometry (Beckman Coulter Gallios). Detailed methods can be found in “*Methods in Malaria Research*” (2013; www.beiresources.org/portals/2/MR4/Methods_In_Malaria_Research-6th_edition.pdf). Merozoite lysis assays were performed as previously described (*14*). Green fluorescent protein–positive (GFP^+^) merozoites were stained with ethidum bromide and incubated with normal sera and IgG and IgM fractions, and the counts of GFP^+^ merozoites were measured by flow cytometry.

### Antibody and complement ELISAs

To assess levels of antibody isotypes targeting merozoites, ELISA was performed following a standard procedure. Ninety-six–well flat bottom MaxiSorp plates (Nunc) were coated with 50 μl of merozoites (2.5 × 10^5^ merozoites/ml) or 50 μl of recombinant antigen (0.5 μg/ml) in PBS overnight at 4°C.

For ELISAs, the following antigens were used: MSP1-42 (42-kDa fragment of MSP1), a gift from C. Long at the National Institutes of Health (His-tagged produced in *Escherichia coli*) (*35*); MSP2 (3D7 allele, full length) and MSP3 [expressed in *E. coli* as previously described (*36*)]; MSP4 and MSP5 (3D7, expressed in *E. coli*), a gift from Ross Coppel at Monash University, Melbourne, Victoria, Australia (*37*, *38*); MSP6 generated as previously described (*36*); Rh2 [expressed in *E.coli* as previously described (*36*)]; and EBA175 and EBA181 [expressed in *E.coli* as previously described (*8*)]. For AMA1 (3D7 allele), the “ecto” domain (amino acids 25 to 546) was expressed following the replacement of the native signal peptide with a tissue plasminogen activator signal peptide and 6-His tag and assessment for potential glycosylation sites (www.cbs.dtu.dk/services/NetNGlyc/) leading to modification at six amino acid residues to prevent potential glycosylation. The PfAMA1 protein sequence was used to generate a DNA sequence that was codon-optimized for mammalian expression and then synthesized (GeneArt). The synthetic genes were supplied in a Puc vector and then cloned into pcDNA 3.1^+^ using Xho 1 and Bam H1 restriction sites. The final plasmid was quantified and used to transfect HEK293F FreeStyle cells (Thermo Fisher Scientific) cultured and transfected following the manufacturer’s protocols. Expressed protein was purified by nickel resin columns (Life Technologies) and washed with 20 mM imidazole/PBS (Sigma-Aldrich), and bound proteins were eluted in several 1-ml fractions in 500 mM imidazole/PBS. Protein size and purity were confirmed by SDS-PAGE. For all ELISAs, plates were washed three times with PBS-Tween 0.05% and blocked with 200 μl of 1% casein (Sigma-Aldrich) in PBS for 2 hours at 37°C. Serum samples (1:250 dilution) or purified IgG or IgM fractions were incubated in 0.1% casein in PBS for 2 hours at room temperature. For detection of antibody isotypes and subclasses, antibody binding was detected with the following antibodies: anti-human IgM (clone HP6083), anti-human IgG1 (clone HP6069), anti-human IgG3 (HP6050; 1:2000) from Invitrogen detected with goat polyclonal anti-mouse IgG HRP conjugate (1:2000; Merck Millipore), or goat polyclonal anti-human IgG HRP conjugate (1:2000; Invitrogen) for 1 hour at room temperature. For detection of complement-fixing antibodies, following incubation with sera or IgG/IgM fraction samples, plates were incubated with 10% normal sera or purified C1q as a complement source. C1q fixation was detected with rabbit anti-C1q antibodies (Dako) and anti-rabbit HRP (Bio-Rad). C3b and membrane attack complex formation was detected with anti-human iC3b or anti-human SC5b-9 (Quidel) and anti-mouse IgG HRP (Merck Millipore). To assess affinity of IgM and IgG, standard ELISAs were performed with the addition of a dissociation step of increasing concentrations of ammonium thiocynate (in PBS) incubated for 20 min at room temperature following incubation with serum samples. TMB substrate (Sigma-Aldrich) was added for 1 hour at room temperature, and the reaction was stopped using 1 M HCl. The OD was read at 450 nm. For ELISA using isolated merozoites, washes were done with PBS without Tween to prevent parasite lysis. Standardization of the assays was achieved using positive control plasma pools on each plate. Background values (wells with no plasma) were subtracted from all values of other wells, and positivity was determined as the means + 3SDs of the OD from naïve plasma samples (Australian residents) included in each assay or for the PNG children longitudinal (Mugil) cohort as the upper 95% CI of naïve plasma samples.

### Statistics

Analysis of the association of IgM/IgG with protection in cohort study was performed with Stata/SE 12.0. Differences in IgM/IgG between groups were assessed by Wilcoxon rank sum tests. For assessment of associations between IgM/IgG and protection, subjects were stratified into three equal-sized tertile groups according to low (including those classified as “negative”), medium, or high IgM and IgG responders, as determined by OD values for each sample. This method allows for a dose-response relationship to be examined while maintaining sufficient numbers of participants in each group to maximize statistical power. Tertile groups were compared for risk of clinical malaria (fever and >5000 parasites/μl) or high-density parasitemia (>5000 parasites/μl) with the Cox proportional hazards model and Kaplan-Meier survival analysis, taking into account the first malaria episode only. Age and location of residence were included in adjusted analysis as these factors were previously identified as potential confounders (*17*). Effect modifications between parameters were investigated by interaction terms; no significant interactions were identified (*P* < 0.1 in all cases). IgM and IgG were also compared between children who were protected (always asymptomatic when infected; *n* = 112) or who were susceptible (had at least one episode of clinical malaria; *n* = 78) during follow up. Logistical regression analysis of protected and susceptible outcomes was adjusted for age and location of residence. This analysis only considered children who had at least one infection during follow-up to ensure that analysis included only children who were exposed to infection.

All other statistical analyses were performed in GraphPad Prism. Wilcoxon matched-pairs signed-rank test was used for comparisons of responses between acute (day 0) and convalescent time points (days 7 and 28) in matched samples. Mann-Whitney *U* test was used to compare responses between different populations (acute versus control) and for invasion inhibition assays. Spearman’s rank correlation coefficient was used to assess correlations between antibody responses and C1q fixation. To assess the relationship between C1q and IgM and IgG subclasses in Sabah individuals, ELISA OD data were transformed into arbitrary units, relative to the highest responder in the cohort.

### Study approval

Written informed consent was obtained from all participants or, in the case of children <18 years of age, from their parents or guardians before inclusion in studies. Ethics approval for the use of human samples in the relevant studies was obtained from the Alfred Human Research and Ethics Committee for the Burnet Institute (435-10, 416-10, and 84-13); the Ethics Committee of the Kenya Medical Research Institute (SSC1131); the Medical Research Advisory Committee of Papua New Guinea (MRAC 98.03); the Medical Research and Ethics Committee, Ministry of Health, Malaysia (NMRR 10 754 6684); the Human Ethics Committee of the Northern Territory Department of Health and Menzies School of Health Research (HREC 10/1431); the Human Research Ethics Committee Melbourne Health (2004.038); and the Human Research and Ethics Committee of the QIMR-Berghofer Institute of Medical Research (P1479).

## Supplementary Material

http://advances.sciencemag.org/cgi/content/full/5/9/eaax4489/DC1

Download PDF

IgM in human immunity to Plasmodium falciparum malaria

## SUPPLEMENTARY MATERIALS

Supplementary material for this article is available at http://advances.sciencemag.org/cgi/content/full/5/9/eaax4489/DC1 Fig. S1. IgM and IgG antibody induction to individual merozoite antigens following primary *P. falciparum* infection in naïve adults. Fig. S2. IgM, IgG1, and IgG3 during clinical *P. falciparum* malaria and following treatment in patients from Sabah. Fig S3. Purification of IgG and IgM fractions. Fig S4. Merozoite lysis with IgM and IgG fractions. Fig S5. C1q-fixing antibodies in Sabah individuals. Table S1. Proportion of responses above positive threshold. Table S2. Cohort characteristics of patients with clinical malaria from Sabah, Malaysia. Table S3. Australia resident returned travelers. Table S4. Prevalence and levels of IgM and IgG to the merozoite surface in the longitudinal cohort of PNG. Table S5. Associations between IgM, IgG, and C1q to the merozoite surface and odds of susceptibility to malaria in PNG children.
