# Supplementary material for: IgM in human immunity to Plasmodium falciparum malaria
Source: Sci Adv. 2019 Sep 25;5(9):eaax4489. doi: 10.1126/sciadv.aax4489 (PMC6760923; doi:10.1126/sciadv.aax4489)
Supplement: http://advances.sciencemag.org/cgi/content/full/5/9/eaax4489/DC1 [file supp_5_9_eaax4489__index.html]

Science Advances | Science AdvancesAAASSearchScience AdvancesMenu

## Supplementary Materials

**This PDF file includes:**

- Fig. S1. IgM and IgG antibody induction to individual merozoite antigens following primary *P. falciparum* infection in naïve adults.
- Fig. S2. IgM, IgG1, and IgG3 during clinical *P. falciparum* malaria and following treatment in patients from Sabah.
- Fig S3. Purification of IgG and IgM fractions.
- Fig S4. Merozoite lysis with IgM and IgG fractions.
- Fig S5. C1q-fixing antibodies in Sabah individuals.
- Table S1. Proportion of responses above positive threshold.
- Table S2. Cohort characteristics of patients with clinical malaria from Sabah, Malaysia.
- Table S3. Australia resident returned travelers.
- Table S4. Prevalence and levels of IgM and IgG to the merozoite surface in the longitudinal cohort of PNG.
- Table S5. Associations between IgM, IgG, and C1q to the merozoite surface and odds of susceptibility to malaria in PNG children.

Download PDF

**Files in this Data Supplement:**

- Adobe PDF - aax4489\_SM.pdf
