# Supplementary material for: IgM in human immunity to Plasmodium falciparum malaria
Source: Sci Adv. 2019 Sep 25;5(9):eaax4489. doi: 10.1126/sciadv.aax4489 (PMC6760923; doi:10.1126/sciadv.aax4489)
Supplement: Download PDF [file aax4489_SM.pdf]

Science Advances
AAAS

advances.sciencemag.org/cgi/content/full/5/9/eaax4489/DC1

Supplementary Materials for

IgM in human immunity to *Plasmodium falciparum* malaria

M. J. Boyle*, J. A. Chan, I. Handayuni, L. Reiling, G. Feng, A. Hilton, L. Kurtovic, D. Oyong, K. A. Piera, B. E. Barber, T. William, D. P. Eisen, G. Minigo, C. Langer, D. R. Drew, F. de Labastida Rivera, F. H. Amante, T. N. Williams, S. Kinyanjui, K. Marsh, D. L. Doolan, C. Engwerda, F. J. I. Fowkes, M. J. Grigg, I. Mueller, J. S. McCarthy, N. M. Anstey, J. G. Beeson*

*Corresponding author. Email: michelle.boyle@burnet.edu.au (M.J.B.); james.beeson@burnet.edu.au (J.G.B.)

Published 25 September 2019, *Sci. Adv.* **5**, eaax4489 (2019)
DOI: 10.1126/sciadv.aax4489

This PDF file includes:

Fig. S1. IgM and IgG antibody induction to individual merozoite antigens following primary *P. falciparum* infection in naïve adults.
Fig. S2. IgM, IgG1, and IgG3 during clinical *P. falciparum* malaria and following treatment in patients from Sabah.
Fig S3. Purification of IgG and IgM fractions.
Fig S4. Merozoite lysis with IgM and IgG fractions.
Fig S5. C1q-fixing antibodies in Sabah individuals.
Table S1. Proportion of responses above positive threshold.
Table S2. Cohort characteristics of patients with clinical malaria from Sabah, Malaysia.
Table S3. Australia resident returned travelers.
Table S4. Prevalence and levels of IgM and IgG to the merozoite surface in the longitudinal cohort of PNG.
Table S5. Associations between IgM, IgG, and C1q to the merozoite surface and odds of susceptibility to malaria in PNG children.

Supplementary Figure S1:

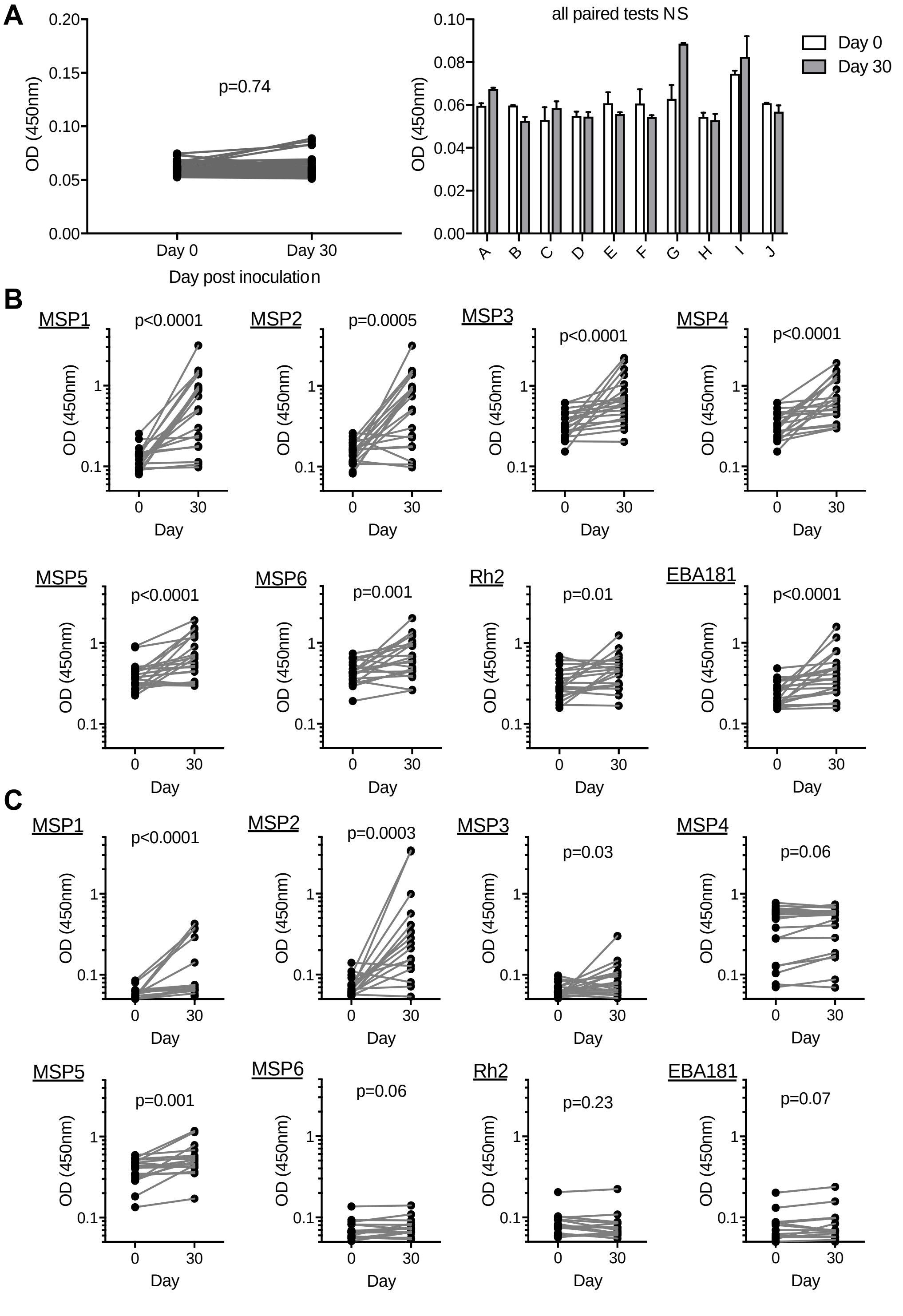

Fig. S1. IgM and IgG antibody induction to individual merozoite antigens following primary *P. falciparum* infection in naïve adults. (A) IgM reactivity to non-malarial protein BSA was tested before (day 0) and after CHMI (day 30), n=40. There was no increase in non-immune IgM following CHMI. A subset of samples are shown individually in right panel. **(B/C)** IgM **(B)** and IgG **(C)** responses to a number of merozoite antigens were assessed at day 30 following infection and compared to day 0 responses prior to infection. Samples used were from the controlled human malaria infection model (n=20).

Supplementary Figure S2:

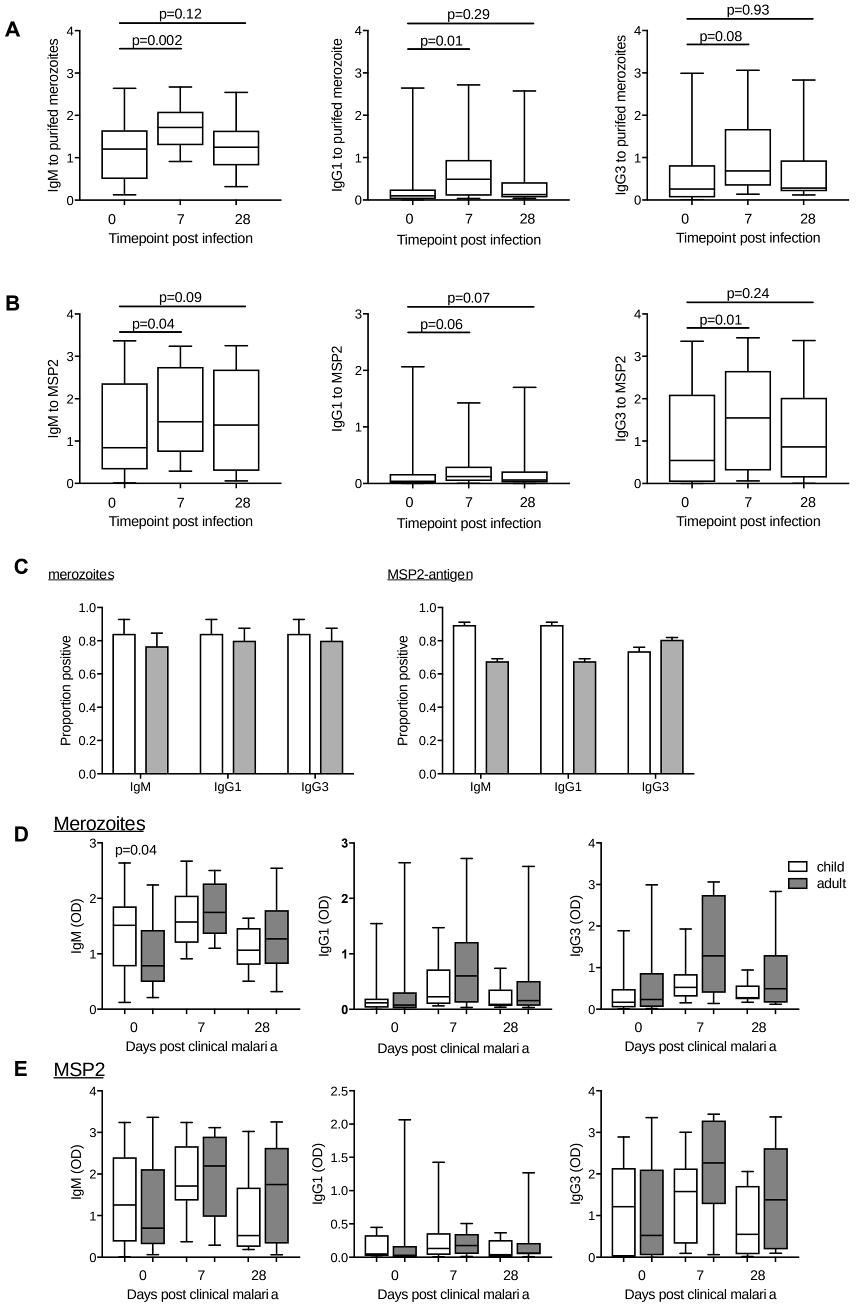

Fig. S2. IgM, IgG1, and IgG3 during clinical *P. falciparum* malaria and following treatment in patients from Sabah. IgM, IgG1 and IgG3 to purified merozoites **(A)** and MSP2 **(B)** were assessed in individuals with clinical malaria (n=50) in patients in Sabah, Malaysia Borneo, and in a subset of patients at 7 (n=26) and 28 days (n=30) after treatment. **(C)** Patients with clinical malaria in Sabah, Malaysian Borneo, were divided into children (≤15 years, n=19) and adults (n=31). IgM, IgG1 and IgG3 to merozoites and MSP2 were assessed during malaria in children, from Sabah, Malaysia Borneo. Seroprevelance was calculated as mean OD ± 3SD of unexposed Australian naïve controls. **(D/E)** IgM, IgG1 and IgG3 to intact merozoites **(D)** and recombinant MSP2 **(E)** were assessed in children and adults during clinical malaria, and at 7 and 28 days after treatment (day 7 children n=14, adults n=12, day 28 children n= 8, adults n=24). Data is ELISA OD.

Supplementary Figure S3:

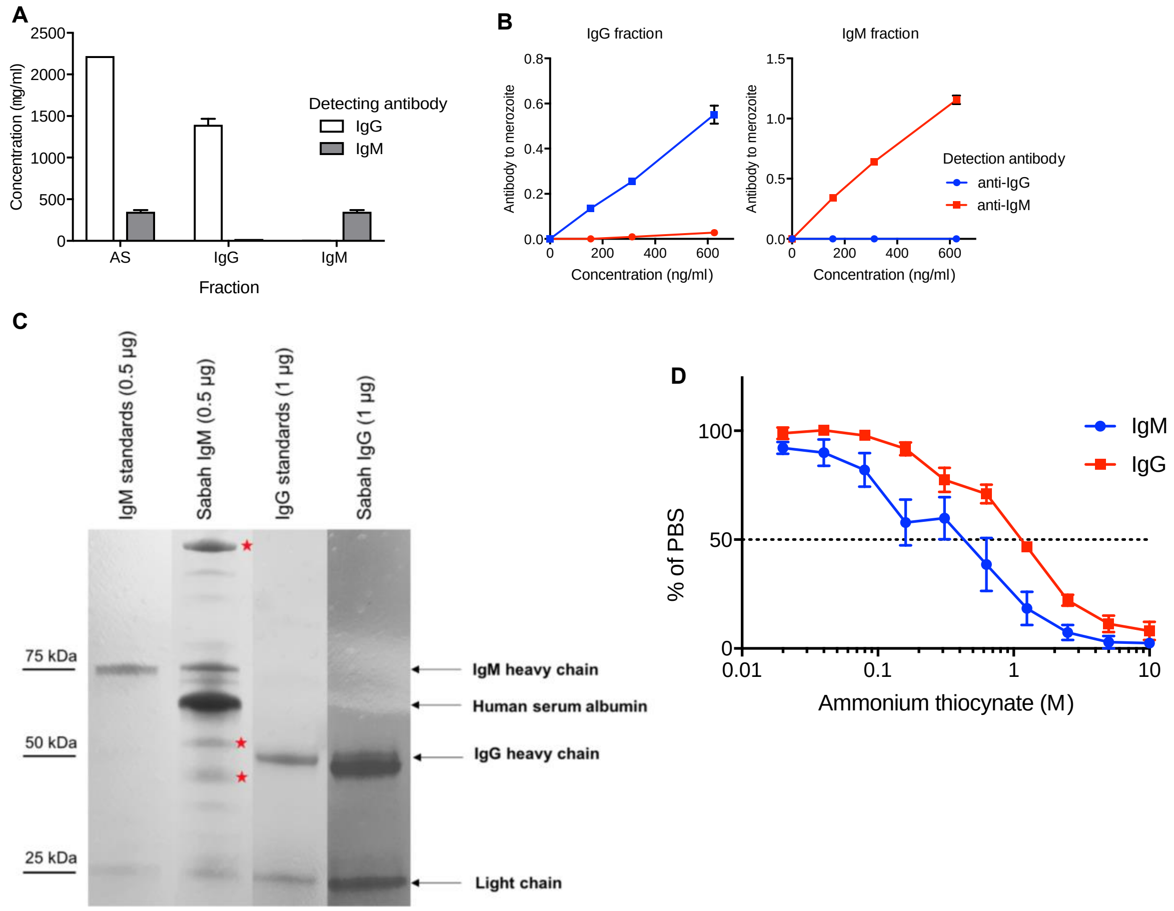

Fig S3. Purification of IgG and IgM fractions. Immunoglobulin from pooled sera from Sabah individuals with a recent *P. falciparum* malaria infection was ammonium sulfate precipitated and then IgG purified via Protein G purification columns. The remaining fraction was considered a 'IgM' fraction. **(A)** The total concentration of IgG and IgM in the ammonium sulfate precipitate (AS) and IgG and IgM fraction was measured via ELISA using IgG and IgM standards. **(B)** Merozoite-specific IgG and IgM detected in each fraction **(C)** The purity of IgG and IgM fractions was assessed via Western blot. Contamination of serum albumin was detected in IgM fraction. Other contaminating proteins (indicated with red star), possibly polymer complex of serum albumins and enzymes, were also observed. However, there was no remaining IgG detected in the IgM fraction, nor IgM in the IgG purified fraction. **(D)** Affinity of IgM and IgG in purified IgM and IgG fractions from Sabah pooled sera was tested by increasing concentrations of ammonium thocynate. Data is mean ± s.e.m. of 3 assays in duplicate.

Supplementary Figure S4:

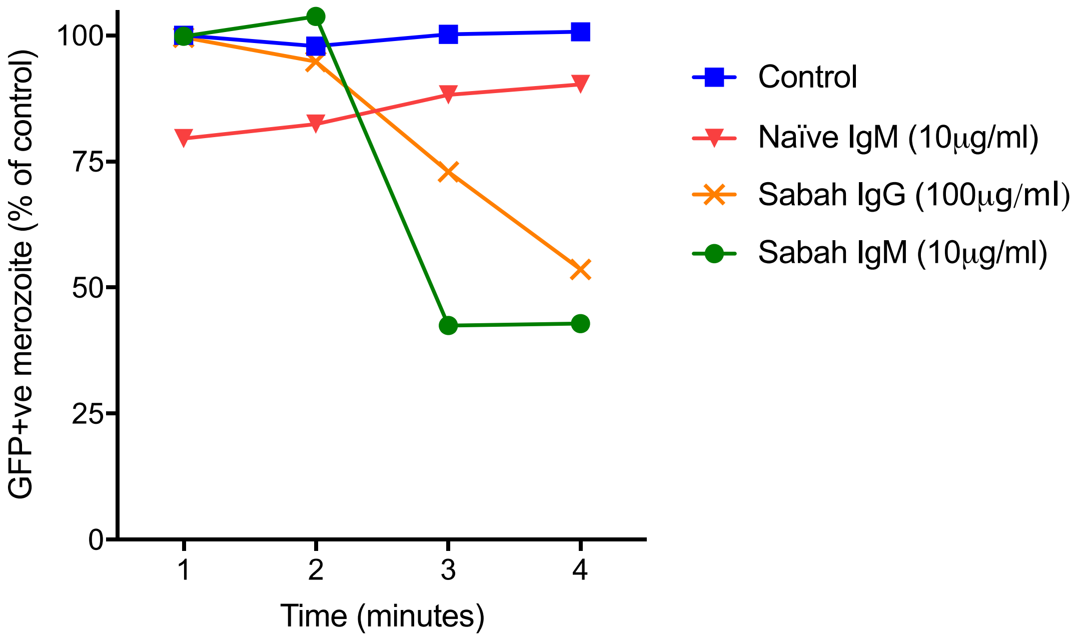

Fig S4. Merozoite lysis with IgM and IgG fractions. IgM (10μg/ml) or IgG (100μg/ml) was incubated with GFP +ve merozoitesand 25% normal sera. Samples were analysed by flow cytometry and GFP+ merozoites assessed over time. Data is expressed as a % of GFP +ve merozoites at 1 minute in control sample. Control is merozoites incubated in heat-inactivated sera.

Supplementary Figure S5:

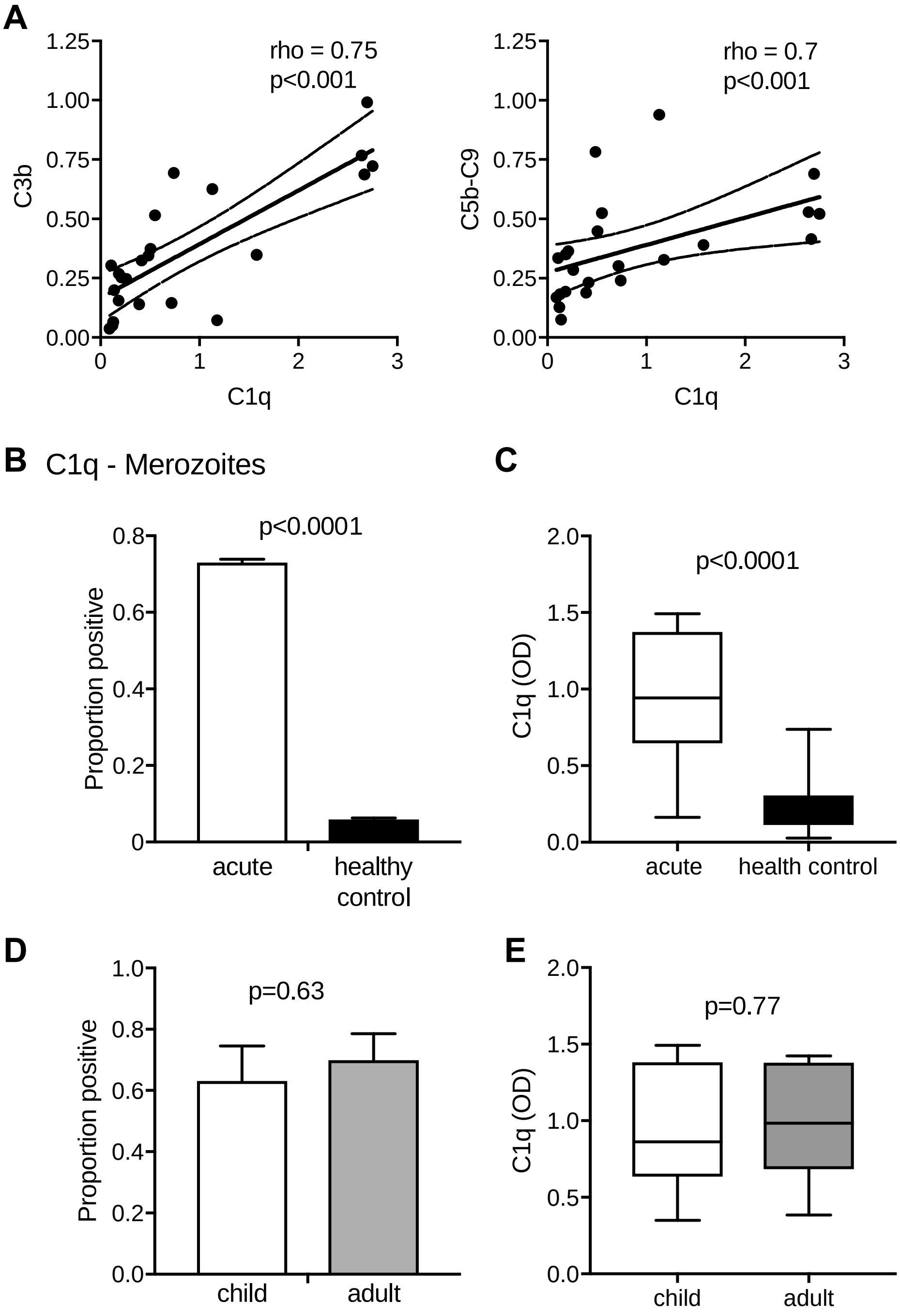

Fig S5. C1q-fixing antibodies in Sabah individuals. (A) C1q, C3b, C5b-C9 deposition to MSP2 antigen was tested in a subset of Sabah individuals (n=23). C1q fixation was correlated with C3b and C5b-C9 deposition, consistent with the activation of classical complement cascade. **(B/C)** C1q fixing antibodies to merozoites were assessed in patients with acute malaria (n=50) and uninfected community controls (n=20) from Sabah, Malaysia Borneo. **(B)** Seropositivity (left panels) is calculated as mean OD ± 3SD of unexposed Australian naïve controls. **(C)** Magnitude of C1q fixing antibodies expressed as OD. **(D/E)** Patients with acute malaria were divided into children (≤15 years, n=19) and adults (n=31). **(D)** Seropositivity (left panels) is calculated as mean OD ± 3SD of unexposed Australian naïve controls. **(E)** Magnitude of C1q fixing antibodies expressed as OD.

Supplementary Tables:

Table S1. Proportion of responses above positive threshold.

Merozoite antigen	Day post infection	Proportion positive [1] (n, %) IgM	IgG	P [2]
MSP2	7	0 (0)	0 (0)	1
	14	10 (25)	4 (10)	0.141
	30	30 (75)	17 (42.5)	**0.006**
AMA1	7	2 (5)	1 (2.5)	1
	14	13 (32.5)	5 (12.5)	0.06
	30	16 (40)	6 (15)	**0.02**
EBA175	7	1 (2.5)	4 (10)	0.356
	14	5 (12.5)	0 (0)	0.06
	30	24 (60)	1 (2.5)	**<0.001**

[1] Positivity cut off calculated from mean+ 3 standard deviation of day 0 responses
[2] Two sample test for equality of proportions without continuity correction

Table S2. Cohort characteristics of patients with clinical malaria from Sabah, Malaysia.

	Total	Children	Adults	p
N	50	19	31	
Male (n, %)	35 (70%)	13 (68%)	22 (71%)	
Age, years (median, IQR)	21 (13-29)	11 (8-13)	24 (21-39)	
Parasites/µl (median, IQR)	12590 (2440-40000)	9924 (2440-51350)	14880 (2390-38025)	0.94

Table S3. Australia resident returned travelers.

Age	Origin[1]	Ethnicity	Parasite density[2]
56	PNG	Melanesian	58000
38	PNG	Caucasian	14484
36	Pakistan	Asian	7040
42	PNG	Caucasian	2600
31	PNG	Caucasian	20000
24	Zimbabwe	Caucasian	180000
25	Ghana	African	29000
19	PNG	Caucasian	67000
24	Kenya	African	81000
30	PNG	Maori	2230

[1] Country where *P. falciparum* infection was acquired
[2] Parasites/µl on presentation

Table S4. Prevalence and levels of IgM and IgG to the merozoite surface in the longitudinal cohort of PNG.

			Age[4]			Enrolment *P. falciparum* parasitemic status[5]		
**Isotype**		**All n = 199**	**<9yrs n=90**	**>=9yrs n=109**	**p**	**PCR- n=65**	**PCR+ n=134**	**p**
**IgM**	**Seropositive[1] %[2]**	179 90%	77 85.6%	102 93.6%	0.06	52 80%	127 94.8%	0.001
	**Median OD [IQR][3]**	0.55 [0.33-0.84]	0.44 [0.27-0.82]	0.6 [0.41-0.89]	0.005	0.37 [0.25-0.56]	0.63 [0.41-0.9]	<0.0001
**IgG**	**Seropositive[1] %[2]**	198 99.5%	89 98.9%	109 100%	0.27	64 98.5%	134 100%	0.15
	**Median OD [IQR][3]**	1.53 [1.1-1.86]	1.44 [0.88-1.83]	1.58 [1.28-1.93]	0.01	1.15 [0.55-1.63]	1.63 [1.36-1.94]	<0.0001

[1] Number of individuals from the cohort with IgM or IgG recognising merozoites' surface, based on the upper 95% CI of naïve plasma samples
[2] %: percent of individuals from the cohort with IgM or IgG recognising merozoites' surface
[3] [IQR]: inter-quartile range
[4] Age: the cohort was stratified by age into two groups: children 9 years of age and younger (≤) or older than 9 years of age
[5] Enrolment *P. falciparum* parasitemic status: PCR- indicates aparasitemic status at enrolment, PCR+ indicates parasitemic status at enrolment, both as determined by PCR

Table S5. Associations between IgM, IgG, and C1q to the merozoite surface and odds of susceptibility to malaria in PNG children.

		uOR [95%CI]	p	aOR [95%CI] age/location	p
**IgM**	LvM	**0.34 [0.16-0.70]**	**0.004**	**0.45 [0.21-0.98]**	**0.043**
	LvH	**0.21 [0.1-0.46]**	**<0.001**	**0.27 [0.12-0.59]**	**0.001**
**IgG**	LvM	**0.40 [0.20-0.83]**	**0.013**	**0.45 [0.21-0.95]**	**0.036**
	LvH	**0.19 [0.09-0.42]**	**<0.001**	**0.22 [0.10-0.53]**	**0.001**
**C1q**	LvM	**0.49 [0.24-1]**	**0.05**	0.57 [0.27-1.18]	0.13
	LvH	**0.09 [0.03-0.21]**	**<0.001**	**0.11 [0.04-0.28]**	**<0.001**

Children who had at least one recorded *P. falciparum* infection were grouped as protected (always asymptomatic when infected), or susceptible (at least one malaria episode in follow-up). Levels of IgM, IgG, and C1q fixing antibodies (previously reported, Boyle et al, Immunity, 2015) were stratified into three equal groups according to low, medium or high levels (defined by tertiles). Unadjusted odd ratios of susceptibility (uOR) and odds ratios adjusted for the predetermined confounders of age and location of residence (aOR) are presented. IgM levels were compared between low-vs-medium (LvM) or low-vs-high (LvH) groups.
